# The critical role of non-contrast chest CT in avoiding thrombolysis catastrophe: A case of acute aortic dissection masquerading as ischemic stroke

**DOI:** 10.1016/j.ijscr.2025.111600

**Published:** 2025-07-02

**Authors:** Xiaoyin Huang, Feiyan Chen, Yadong Wu

**Affiliations:** a Department of Emergency, Sir Run Run Shaw Hospital, Medical School of Zhejiang University, Hangzhou 310000, China; b Department of Catheterization room, Sir Run Run Shaw Hospital, Medical School of Zhejiang University, Hangzhou 310000, China

**Keywords:** Acute aortic dissection, Ischemic stroke, Chest CT, Thrombolysis

## Abstract

**Introduction and importance:**

Aortic dissection (AD) complicating acute ischemic stroke poses significant therapeutic challenges, particularly regarding thrombolytic contraindications that may substantially increase mortality risk. This underscores the critical need for expedited differential diagnosis in such clinical scenarios.

**Case presentation:**

An 80-year-old male presented with acute-onset left-sided hemiplegia and unresponsiveness.

**Clinical discussion:**

Initial cerebral neuroimaging demonstrated a substantial ischemic penumbra (136.2 ml Tmax >6 s) in the right middle cerebral artery (MCA) territory, with a core infarction volume of 13.6 ml. Emergency intravenous thrombolysis with alteplase was administered for presumed acute ischemic stroke, followed by mechanical thrombectomy via femoral artery access. Retrospective analysis of preoperative chest Computed tomography (CT) imaging revealed ascending aortic dilation. Subsequent emergency aortic CTA confirmed Stanford Type A dissection extending from the aortic root to the first lumbar vertebral level. Despite comprehensive risk counseling, the patient's family ultimately elected for discharge against medical advice. Telephone follow-up at 30 days post-discharge confirmed patient demise.

**Conclusion:**

This case highlights two critical clinical considerations: Subtle radiographic manifestations of AD in stroke patients may lead to diagnostic delays; the hyper-dense crescent sign on non-contrast cranial CT warrants heightened clinical suspicion for concurrent aortic pathology.

## Highlights

• **Chest CT Saves Lives:** Routine integration of non-contrast chest CT into stroke protocols for high-risk patients (hypertension, NIHSS >10, unexplained ECG anomalies, history of aortic disease) can prevent lethal thrombolysis errors.
• **Time for Guideline Reform**: Current stroke pathways must prioritize aortic screening in elderly populations, balancing the "time-is-brain" paradigm with vascular safety.
• Our case suggests that patients considering stroke **should have a simultaneous non-contrast chest CT in addition to head CT.**

## 1 Introduction and importance

Acute aortic dissection (AD) complicated by ischemic stroke is a rare but fatal condition, with mortality exceeding 80 % if misdiagnosed [1]. The diagnostic challenge arises from overlapping symptoms: up to 30 % of AD patients present with neurological deficits, such as hemiplegia and altered consciousness, that mimic acute ischemic stroke (AIS), often delaying recognition of the underlying aortic emergency [2,3]. Furthermore, intravenous thrombolysis (IVT)—the cornerstone of AIS treatment—is contraindicated in AD due to the risk of dissection extension or rupture, leading to hemorrhagic shock [4,5].

This case report describes an aortic dissection masquerading as acute stroke, ultimately diagnosed post-thrombolysis/thrombectomy. Retrospective review revealed a dilated ascending aorta on the non-contrast chest CT, underscoring its important role in detecting stroke-mimicking dissections. This work has been reported in line with the SCARE criteria [6,7].

## 2 Case presentation

**Fig. 1:**
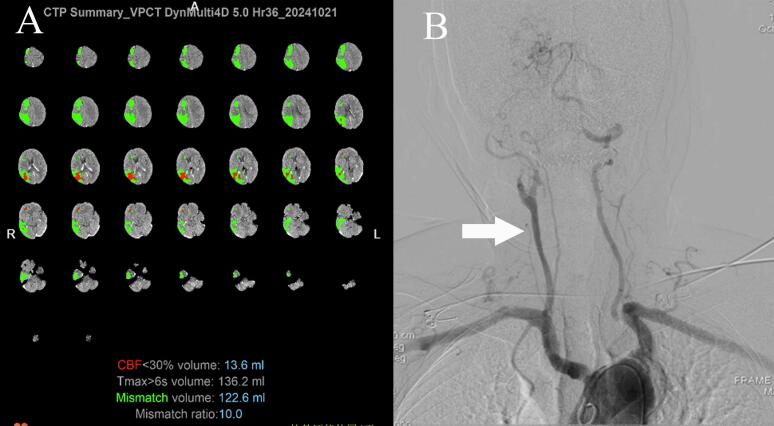
(A) Computed tomography perfusion (CTP) scan identified a significant ischemic penumbra (136.2 ml Tmax >6 s) in the right middle cerebral artery (MCA) territory, with a core infarction volume measuring 13.6 ml; (B) Angiographic finding showed complete occlusion of the right middle cerebral artery (MCA) M1 segment (white arrow).

On October 25, 2024 at 07:30, an 80-year-old male with a history of untreated hypertension, atrial fibrillation, and coronary artery disease was found unconscious in a field, exhibiting left-sided hemiplegia and unresponsiveness. Upon admission to the emergency department at 09:00, initial vital signs included a blood pressure of 132/86 mmHg, heart rate of 75 bpm, and a National Institutes of Health Stroke Scale (NIHSS) score of 16. Non-contrast cranial CT imaging revealed bilateral periventricular ischemic changes without acute hemorrhage. Subsequent CT perfusion (CTP) studies identified a significant ischemic penumbra (136.2 ml Tmax >6 s) in the right middle cerebral artery (MCA) territory, with a core infarction volume of 13.6 ml (Fig. 1A). Emergency intravenous thrombolysis with alteplase was initiated for presumed acute ischemic stroke. During thrombolysis therapy, the patient developed chest discomfort, accompanied by hypotension (85/50 mmHg) and progressive deterioration in consciousness. Electrocardiography demonstrated atrial fibrillation with complete heart block (ventricular rate 42 bpm) and T-wave inversions in inferior (II, III, aVF) and lateral (V4-V6) leads. Cardiology consultation excluded acute coronary syndrome but confirmed hemodynamic instability requiring urgent intervention.

Under general anesthesia, mechanical thrombectomy was performed via femoral artery access. Initial angiography showed: 1.Complete occlusion of the left common carotid artery (CCA) with indistinct ostium; 2.Moderate-to-severe stenosis at the origin of the left dominant vertebral artery; 3.Retrograde dissection-related contrast enhancement at the right internal carotid artery (ICA) origin with delayed distal flow; 4.Complete occlusion of the right middle cerebral artery (MCA) M1 segment (Fig. 1B). A 4 × 20 mm stent retriever deployed through an intermediate catheter system successfully extracted two 1 cm red thrombi after two retrieval attempts. Post-procedural angiography confirmed complete MCA recanalization (mTICI 3) without contrast extravasation. Postoperative cranial CT revealed no intracranial hemorrhage. The patient was transferred intubated to the intensive care unit (ICU) under mechanical ventilation (FiO2 40 %, SpO2 94 %; Ventilator settings: A/C mode, PAP 13, f 13, TVE 450, I:E 1:2) at 14:00. Neurological deficits persisted, including left-sided facial droop, hemiplegia (left limb power 0/5), and hypertonia, consistent with large right MCA infarction.

**Fig. 2:**
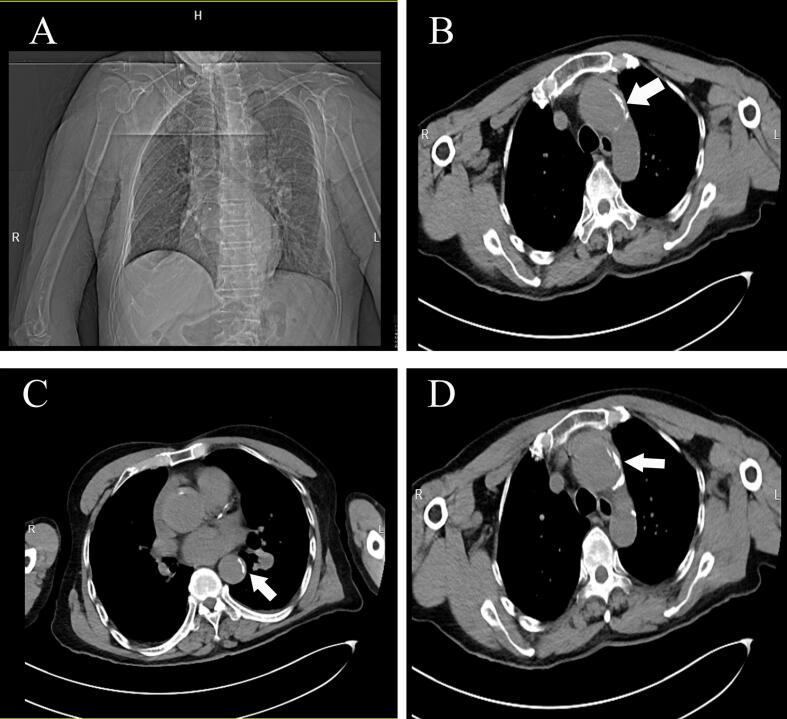
Non-contrast chest computed tomography (CT) scan shows eccentric wall thickening with hyper-density (“crescent sign”) (white arrows).

**Fig. 3:**
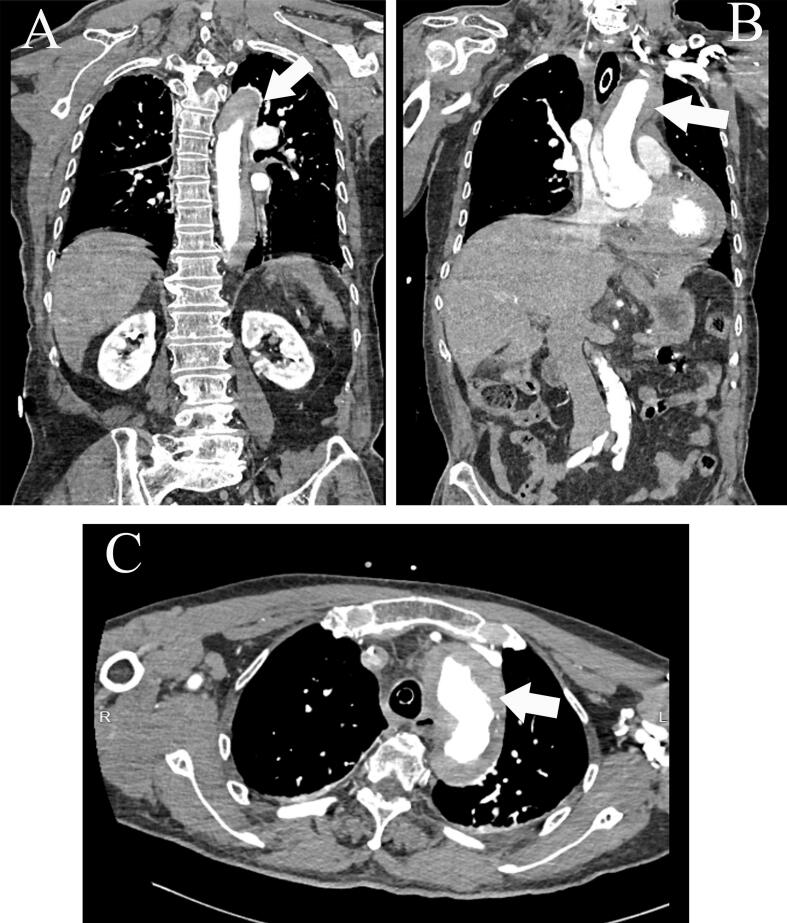
Aortic CTA confirmed Stanford Type A dissection extending from the aortic root to the L1 vertebral level.

Retrospective review of preoperative chest CT imaging identified ascending aortic dilation (42 mm, Fig. 2A), eccentric wall thickening with hyper-density (“crescent sign”, Fig. 2B-D, white arrows), and mediastinal widening. Subsequent aortic CTA confirmed Stanford Type A dissection extending from the aortic root to the first lumbar vertebral level, accompanied by pericardial effusion (20 mm, Fig. 3A-C, white arrows). Laboratory findings included elevated D-dimer (1.00 μg/mL) but normal cardiac troponin and coagulation profiles.

Upon diagnosis, a multidisciplinary team—including cardiac surgery, thoracic surgery, interventional radiology, critical care, and neurology—was immediately convened at 15:40. Corrective measures for coagulopathy were initiated with both fresh frozen plasma transfusion and fibrinogen concentrate administration. The team urgently recommended cardiopulmonary bypass surgery for aortic dissection repair. Given the family's refusal of interventions, no surgical or medical interventions was implemented prior to discharge.

Given the patient's advanced age, bilateral cerebral infarction, and high surgical risk, the family declined aortic intervention after extensive counseling. After fully acknowledging the critical prognosis with anticipated rapid clinical deterioration and high mortality risk, they insisted on discharge against medical advice and left the hospital at 19:00. During a telephone follow-up 30 days later, the family reported that the patient had died on the morning of October 26.

## 3 Clinical discussion

The coexistence of acute aortic dissection (AD) and ischemic stroke presents a critical diagnostic dilemma. Up to 30 % of AD patients lack classic symptoms such as chest or back pain, particularly in elderly populations, leading to misdiagnosis as isolated stroke [1,8]. In this case, neurological deficits (unconsciousness, hemiplegia) masked aortic pathology, delaying AD suspicion until incidental chest CT findings emerged. The hemodynamic instability during thrombolysis (hypotension, heart block) likely resulted from aortic dissection progression and cardiac tamponade, exacerbated by alteplase-induced coagulopathy.

Non-contrast cranial CT, while essential for excluding cerebral hemorrhage, fails to detect aortic abnormalities, and reliance on clinical symptoms such as chest pain is insufficient in elderly patients with sensory impairment. The urgency of thrombolysis further complicates management: IV alteplase, administered here under stroke protocol, carries a 90 % mortality risk in undiagnosed AD due to dissection extension or rupture. This patient's post-thrombolysis hemodynamic instability (hypotension, complete heart block) exemplifies the peril of delayed AD recognition. Current guidelines, which do not mandate aortic screening before IVT unless overt symptoms exist, inadequately address high-risk subgroups.

In this case, the absence of characteristic symptoms (e.g., chest/back pain) contributed to delayed recognition of aortic dissection, underscoring the need for objective screening tools in high-risk stroke patients. Non-contrast chest CT emerged as the lifesaving tool in this case [9]. The “crescent sign”, a hyper-dense aortic wall indicating intramural hematoma, provided the first clue to AD. Wang J et al. reported a patient with stroke-mimicking dissection diagnosed via same-admission CTA. Underwent emergency surgical repair with survival, contrasting our case and demonstrating that early aortic screening enables life-saving intervention [10].

For high-risk patients (NIHSS >10, refractory hypertension, or ECG anomalies), we propose a two-step imaging protocol: (1) concurrent non-contrast chest CT during initial stroke evaluation to identify aortic dilation (>40 mm) or wall abnormalities; (2) immediate aortic CTA if anomalies are detected. This approach balances the “time-is-brain” imperative with vascular safety, adding <5 min to the workflow. Multidisciplinary coordination was pivotal: cardiologists stabilized hemodynamics, neurologists prioritized cerebral perfusion, and radiologists interpreted subtle aortic signs. This case advocates for guideline updates to integrate aortic screening into stroke protocols, particularly for hypertensive elderly populations. Non-contrast chest CT is recommended for stroke patients with NIHSS >10 plus any of: Refractory hypertension (SBP >180 mmHg despite treatment); Unexplained ECG anomalies (e.g., heart block, ischemic changes); History of aortic disease.

## 4 Conclusion

This case highlights two critical lessons: **Chest CT Saves Lives:** Routine integration of non-contrast chest CT into stroke protocols for high-risk patients (hypertension, NIHSS >10, unexplained ECG anomalies, history of aortic disease) can prevent lethal thrombolysis errors. **Time for Guideline Reform**: Current stroke pathways must prioritize aortic screening in elderly populations, balancing the “time-is-brain” paradigm with vascular safety.

## Author contribution

The two authors Feiyan Chen and Xiaoyin Huang contributed equally to this work and should be considered co-first authors.

## Consent

Written informed consent was obtained from the patient for publication of this case report and accompanying images. A copy of the written consent is available for review by the Editor-in-Chief of this journal on request.

## Ethical approval

This study is exempt from ethical approval at our institution.

## Guarantor

Yadong Wu.

## Research registration number

None.

## Sources of funding

None.

## Declaration of competing interest

The authors declare that there is no conflict of interest for the publication of this article.
